# Economic impact of a more extensive use of FENO testing on the Italian population with asthma

**DOI:** 10.1186/s12931-023-02437-y

**Published:** 2023-06-02

**Authors:** Carla Rognoni, Carlo Milano, Enrico Heffler, Matteo Bonini, Luisa Brussino, Giovanna Elisiana Carpagnano, Fabio Luigi Massimo Ricciardolo, Francesco Costa, Patrizio Armeni

**Affiliations:** 1 Centre for Research on Health and Social Care Management (CERGAS), SDA Bocconi School of Management, Bocconi University, Milan, Italy; 2 Personalized Medicine, Asthma and Allergy, IRCCS Humanitas Research Hospital, Rozzano, MI Italy; 3 Department of Biomedical Sciences, Humanitas University, Pieve Emanuele, MI Italy; 4 Department of Cardiovascular and Thoracic Sciences, Catholic University of the Sacred Heart, Rome, Italy; 5 SSDDU Immunologia, Ospedale Mauriziano, Allergologia, Torino, Italy; 6 Policlinico di Bari, UOC di Malattie dell’Apparato Respiratorio, Università Aldo Moro, Bari, Italy; 7 Department of Clinical and Biological Sciences, University of Torino, Torino, Italy; 8 Severe Asthma and Rare Lung Disease Unit, San Luigi Gonzaga University Hospital, Orbassano, Torino Italy

**Keywords:** Fractional exhaled nitric oxide testing, Cost of illness, Asthma, Burden

## Abstract

**Background:**

Asthma is a common chronic inflammatory airway affecting over 260 million people worldwide, and characterized, in the large majority of cases, by the so-called “type 2 inflammation”. Fractional exhaled nitric oxide (FE_NO_) testing is noninvasive point-of-care tool to assess type 2 inflammation and therefore improve asthma management. It has been suggested to determine eligibility for a specific biologic therapy and predict likelihood to respond. The aim of this study was to estimate the overall economic impact of an extensive use of FE_NO_ testing on the Italian population with asthma, including extra costs of testing and savings generated by more appropriate prescriptions, increased adherence and lower frequency of exacerbations.

**Methods:**

A cost of illness analysis was firstly performed to estimate the yearly economic burden from the National Healthcare Service (NHS) perspective in Italy of the management of asthmatic patients with standard of care (SOC) according to the application of GINA (Global Initiative for Asthma) guidelines; then, we evaluated the changes in the economic burden in patient management by introducing FE_NO_ testing into clinical practice. The cost items considered were: visits/exams, exacerbations, drugs, management of adverse events caused by short-term oral corticosteroids use. Efficacy of FeNO test and SOC is based on literature evidence. Costs refer to published data or Diagnosis Related Group/outpatient tariffs.

**Results:**

Considering one asthma visit every 6 months, the total yearly cost for the management of patients with asthma in Italy is 1,599,217,876€ (409.07€ per patient), while for FE_NO_ testing strategy this figure is 1,395,029,747€ (356.84€ per patient). An increased utilization rate of FE_NO_ testing from 50 to 100% of patients may lead to savings for the NHS from about 102 to 204 million € compared to SOC.

**Conclusions:**

Our study showed that FeNO testing strategy may improve the management of asthmatic patients leading to significant savings for the NHS.

## Introduction

Asthma is a chronic inflammatory airway disease that affected about 262 million people in 2019 and caused 461,000 deaths [1]. Asthma is characterized by chronic inflammation associated with variable bronchial obstruction and airway hyperreactivity. Typical clinical presentations include recurrent dyspnea, wheezing, chest tightness and dry cough. Asthma is associated with a substantial burden on quality of life [2], frequently interferes with daily activities and may lead to life-threatening exacerbations.

It is important to underline the distinction between different types of asthma exacerbations depending on their severity defined on the basis of the intensity of therapeutic interventions required.

Mild exacerbations require only reinforcement of prescribed inhaled therapy; moderate exacerbations require a short period of therapy with oral corticosteroids; severe exacerbations require access to the emergency room or hospitalization [3, 4].

Asthma treatment is mainly based on the use of inhaled corticosteroids (ICS), associated or not with long-acting beta2-agonists (LABA) often in combination therapy; other drugs (leukotriene-receptor antagonists, long-acting muscarinic agents, etc.) can be added if optimal control is not achieved with ICS/LABA identifying patients with greater disease severity. Patients not adequately controlled with high dose of ICS plus another controller or using oral corticosteroids (OCS) for more than 6 months per year, are defined as severe asthmatics and should be evaluated for biological treatment with monoclonal antibodies such as omalizumab, mepolizumab, benralizumab and dupilumab [5]. Biological treatments are reimbursed under specific criteria defined by AIFA [6].

Standard of care consists in various follow up visits (frequency range 3–12 months) where a chest examination and spirometry are commonly performed (according to GINA guidelines) in order to find the best management plan and monitor asthma [7]. Asthma worsenings can be managed by patients or by clinicians in home care or hospital setting, depending on their severity. The main asthma complications include: (i) signs and symptoms that interfere with sleep, (ii) work and other activities, (iii) sick days from work or school during asthma exacerbations, (iv) emergency room visits and hospitalizations for severe asthma exacerbations, (v) side effects from long-term use of some medications used to control severe asthma. Proper treatment makes a crucial difference in preventing both short- and long-term complications caused by asthma. It is also useful to identify diseases such as rhinosinusitis (mainly with nasal polyps), bronchiectasis and gastro-esophageal reflux, or conditions like overweight/obesity acting as comorbidities that may underlie asthma or influence its development [8].

The large majority of patients with asthma (50–70%) are characterized by having the so-called “type-2” airway inflammation that involves several cells, such as T-helper 2 cells (Th2), Innate Lymphoid Cells type 2 (ILC-2), eosinophils, mast-cells, and cytokines interleukin (IL)-4, IL-5 and IL-13 [9]. IL-4 and IL-13 cause upregulation of the expression of epithelial inducible nitric oxide synthase (iNOS), a process which is corticosteroid sensitive. Thus, exhaled nitric oxide is a direct signal of the Type- 2 mediated, pro-inflammatory cytokine mechanisms of central importance in the pathophysiology of Type-2 airway inflammation [10–12].

Studying the specific type of patient’s airways inflammation can help doctors making the right diagnosis, find the best management plan and better monitor asthma. Fractional exhaled nitric oxide (FE_NO_) is a noninvasive, point-of-care, easily performed biomarker of airway inflammation used in both the assessment and management of asthma, as it is strongly associated with type 2 inflammation [12]. It’s assessment in patients with asthma may improve asthma management, determine eligibility for a specific biologic therapy and predict likelihood to respond to corticosteroids and to monoclonal antibody anti-IL4-receptor alpha (dupilumab). Indeed, high FeNO predicts risk of exacerbations and lung function decline, so the use of the test could help physicians in better managing patients and controlling the disease.

Few studies have been published in the literature evaluating the cost-effectiveness of FeNO testing compared to the management of patients according to published guidelines. Berg and Price [13, 14] shared the same model for German and UK perspectives considering a 1-year time horizon; in Germany, in mild to severe patients, asthma management with FeNO measurement instead of standard guidelines resulted in cost-savings of 30€ per patient per year. In a more severe population, management with FeNO measurement would save 160€ per patient. In UK, asthma management using FeNO testing instead of lung function testing resulted in annual cost-savings of 341£ and 0.06 quality-adjusted life-years gained for patients with mild to severe asthma and cost-savings of 554£ and 0.004 quality-adjusted life-years gained for those with moderate to severe asthma. Sabatelli and colleagues [15] showed that adding FE_NO_ to standard asthma care may save 62.53€ per patient-year in the adult population and may improve quality-adjusted life years by 0.026 per patient-year. The budget impact analysis revealed a potential net yearly saving of €129 million if FeNO monitoring had been used in primary care settings in Spain [15].

The aim of the present study was to estimate the overall economic impact of an extensive use of FeNO testing on the Italian population, including extra costs of testing and savings generated by more appropriate prescriptions, increased adherence and lower frequency of exacerbations/lung function impairment.

## Methods

A cost of illness (COI) analysis [16] was performed to describe the different types of costs related to asthma in the Italian population. The objective of the COI analysis was firstly to estimate the yearly economic burden from the Healthcare Service perspective in Italy of the management of asthmatic patients according to standard of care (SOC) that refers to the application of the most recent GINA guidelines [7]; secondly, we evaluated the changes in the economic burden of managing these patients considering the introduction in clinical practice of the use of FeNO testing. A MSExcel model has been developed and the cost items considered were: (1) Visits/exams; (2) Exacerbations (non-severe and severe requiring a hospitalization); (3) Drugs (inhaled corticosteroids/combinations and other treatments); (4) Management of adverse events caused by the use of short-term oral corticosteroids.

An advisory board was organized on 14th April 2022 with the participation of five key opinion leaders (KOLs) in the field to discuss the aspects related to the implementation of the model and gain clinical inputs.

### Epidemiological data

The analysis considered the current Italian population (59,236,213) [17] to which an asthma prevalence of 6.60% has been applied [18]. Considering a mortality of 434 asthma patients per year (assumed to occur at mid-year), the considered population of Italian asthmatic patients was composed by 3,909,590 patients.

#### Efficacy of FeNO testing

The literature reports different randomized controlled trials (RCTs) comparing FeNO testing with standard of care (according to clinical guidelines) for the management of patients with moderate to severe asthma and these studies highlighted the benefits of FeNO testing. The paper by Green and colleagues [19] showed that patients managed with FeNO test had significantly fewer severe asthma exacerbations than patients managed following standard British Thoracic Society asthma guidelines (35 vs. 109; p = 0·01); moreover, significantly fewer patients were admitted to the hospital with asthma (1 vs. 6, p = 0·047) showing a decrease of 83% with FeNO strategy. The management of patients with FeNO testing compared to standard of care reported a relative risk reduction of exacerbations of 29% [20] and a reduction in inhaled corticosteroid (ICS) dose of 42% [21] (370 µg per day for the FeNO group vs. 641 µg per day for the control group). Table 1 summarizes the model inputs.

### Healthcare resource use and frequency of events

For SOC we considered one specialist visit every 6 months during which a spirometry is performed and pharmacological therapy recommended. For FeNO strategy the same assumptions were considered, with the inclusion of the test. The KOLs stated that a follow-up time shorter than 6 months between two visits did not represent the clinical practice in Italy.

The total number of exacerbations per year was estimated by data reported in the literature. One paper reported the rate of exacerbations per patient per year from retrospective cohort studies in UK (0.11) and US (0.16) [22]. A mean value was applied for Italy thus estimating 527,765 exacerbations per year. The number of serious exacerbations requiring hospitalization for adults and children was retrieved from two Italian publications [23, 24] and were 10,028 and 6,292, respectively.

In case of asthma exacerbation (severe or non-severe) it was assumed that patients are administered a short course of oral corticosteroids according to GINA guidelines [7]. Corticosteroids are powerful anti-inflammatory drugs that may increase however the risk of serious acute complications such as infection, venous thromboembolism, fracture, as well as chronic diseases such as diabetes mellitus, blood hypertension and osteoporosis. In the model we referred to the literature [25] reporting the incidence rates (per person year at risk) of few adverse events like sepsis (0.0018), venous thromboembolism (0.0046) and fractures (0.0214). As FeNO testing reduces the frequency of exacerbations, this implies a reduction of the frequencies of adverse events associated to short course of oral corticosteroids.

### Costs

For specialist visit, spirometry and FeNO test, we applied the National reimbursement tariffs for outpatients’ services. For hospitalizations due to serious exacerbation we applied the DRG tariffs; for adults we calculated the weighted mean of the reimbursement tariff for DRG 096 and 097 (8,718 and 8,402 cases in 2019, respectively) equal to 2,191€, while for children we referred to the reimbursement tariff for DRG 098. The cost for the management of a non-serious exacerbation was retrieved from a recent Italian study that reported a value of 330€ [26]. The cost for the management of adverse events following short course of oral corticosteroids was retrieved from economic evaluation studies related to the Italian context [27–29].

Concerning treatments, we estimated the overall costs for ICS, possible associations and other drugs starting from statistics provided by the Italian observatory on the use of medicines. We referred to the most recent data available reporting the detailed classification for the different treatments [30]. Table 1 summarizes the cost inputs.

**Table 1:** Model parameters

Description	Value	Reference
Clinical parameters		
Relative risk reduction of hospitalization for serious exacerbation	83%	Green 2002 [19]
Relative risk reduction of non-serious exacerbations	29%	Jayaram 2006 [20]
Reduction in ICS dose	42%	Smith 2005 [21]
Rates of adverse events by short term use of oral corticosteroids (per person year at risk)		
Sepsis	0.0018	Waljee 2017 [25]
Venous thromboembolism	0.0046	Waljee 2017 [25]
Fractures	0.0214	Waljee 2017 [25]
Healthcare resource use		
Period between two follow-up visits for standard of care strategy (months)	6.00	GINA guidelines [7]
Period between two follow-up visits for FeNO strategy (months)	6.00	GINA guidelines [7]
Costs		
Asthma visit	16.20€	National tariff 89.01.L
FE_NO_ test	23.20€	National tariff 93.99.4
Spirometry	24.00€	National tariff 89.37.1
Severe asthma exacerbation	2,537€	DRG 096 (age > 17 years, with complications)
	1,832€	DRG 097 (age > 17 years, without complications)
	1,538€	DRG 098 (age < 18 years)
Non-severe asthma exacerbation	330€	Pugliese 2020 [26]
Sepsis	29,985.08€	Lucioni 2001 [29]
Venous thromboembolism	1,570.24€	Gussoni 2013 [27]
Fractures	6,311.40€	Degli Esposti 2011 [28]
ICS and associations for asthma (total cost for Italy)	663,200,000€	OSMED 2018 [30]
Other drugs for asthma (total cost for Italy)	317,500,000€	OSMED 2018 [30]

### Analyses

For the analyses we compared the SOC scenario, which considers the management of asthma patients according to GINA guidelines, to two different scenarios in which an increased use of FeNO testing, from 50 to 100%, was considered. For each scenario the total cost for the management of patients in Italy was assessed.

For SOC scenario the total cost for asthma hospitalizations was calculated multiplying the number of serious exacerbations by the cost for the hospitalization due to serious exacerbation, distinguishing between adults and children. Analogously, the cost for non-serious exacerbations has been calculated multiplying the number of serious exacerbations by the cost for the management of a single exacerbation. The total cost for the management of adverse events due to short course of OCS has been calculated multiplying the cost for the management of a single event by the number of events; the model estimated 950 sepsis, 2,428 venous thromboembolism events and 11,294 fractures. Concerning the visits, the cost for a specialist visit and of a spirometry have been taken into consideration.

For FE_NO_ testing scenarios, a 83% relative risk reduction of hospitalization for serious exacerbation has been applied to the number of hospitalizations related to SOC. In the same way, a 29% relative risk reduction of non-serious exacerbations and 42% reduction in ICS dose have been considered for FE_NO_ strategy compared to SOC. Concerning the use of ICS, we assumed that a reduction in dose is reflected into a reduction in cost. In these scenarios the cost for the FE_NO_ Testing has been included for each visit.

A scenario analysis has been conducted by considering for both strategies a specialist visit performed every three months according to the lower limit recommended by GINA guidelines.

## Results

For SOC scenario the total cost for asthma hospitalizations per year was 31,648,508€ while the cost for the management of non-serious exacerbations was 168,976,433€. The total drug use was estimated in 980,700,000€ and the cost for the management of adverse events was 103,579,340€. The cost for specialist visits and spirometries was 314,313,594€. All the cost components lead to a total yearly cost for the management of patients with asthma in Italy of 1,599,217,876€ that translates into 409.07 € per patient.

The scenario which considers the use of FeNO testing in the clinical practice highlights a total cost for asthma hospitalizations per year was 5,380,246€ and a cost for the management of non-serious exacerbations of 119,973,268€. The reduction of ICS dose leads to a total cost for drugs of 702,156,000€, while the reduction of the frequency of adverse events shows a cost of 71,811,729€. Total yearly costs for visits, including spirometries and FE_NO_ tests, lead to costs respectively of 126,663,687€, 187,649,907€ and 181,394,910€. The total cost per year for the management of patients in this scenario is 1,395,029,747€ that corresponds to 356.84€ per patient.

**Fig. 1:**
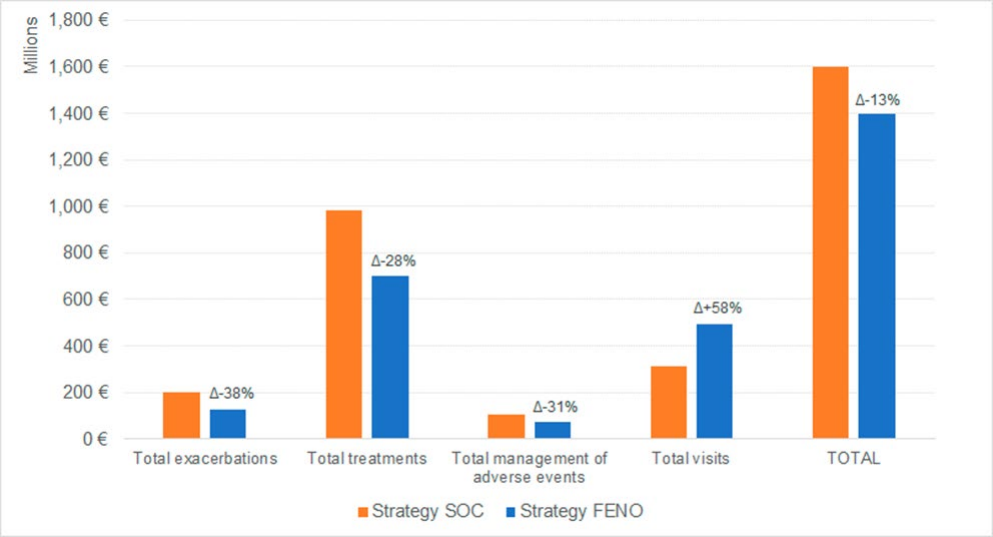
Summary of costs for the main categories for SOC and FE_NO_ test strategies

Table 2 shows the detail for the different cost components; except the cost for FeNO testing, which is considered only for the related strategy, the management of exacerbations requiring hospitalization is the category whose cost showed the greater variation (-83%) for FE_NO_ strategy compared to SOC. The overall difference between the two considered strategies is 13% in favor of FE_NO_ testing. Figure 1 reports the costs for the different categories for the two considered strategies with variations for FE_NO_ test strategy compared to SOC.

**Fig. 2:**
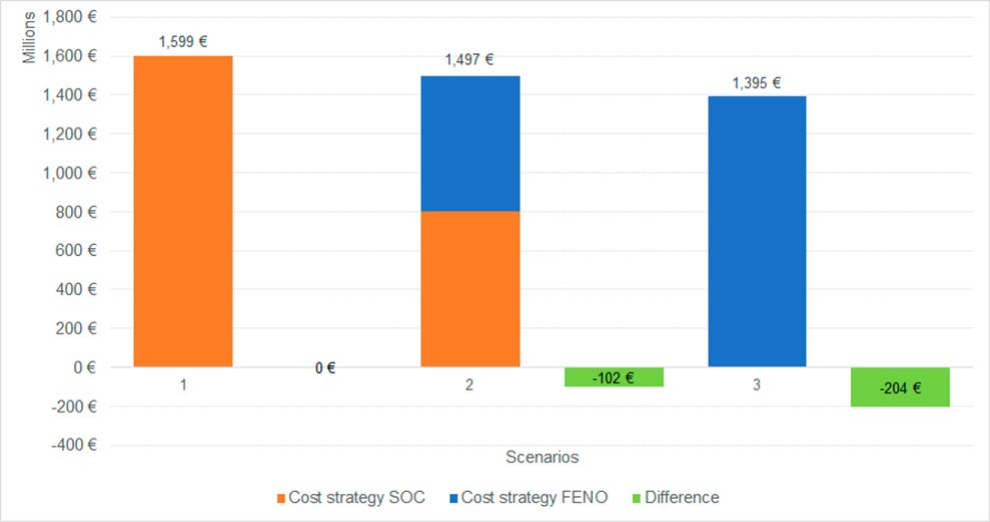
Costs for the different scenarios considered (1: 100% SOC, 2: 50% SOC and 50% FE_NO_ testing, 3: 100% FE_NO_ testing). Differences are related to the comparison versus scenario 1

Table 3 summarizes the total yearly costs for the management of asthma patients in Italy according to different scenarios considered: (1) all patients managed with SOC; (2) 50% of patients managed with SOC and the remaining with the addition of FE_NO_ testing; (3) all the patients managed with FeNO testing strategy. Scenarios 2 and 3 show savings of about 102 and 204 million €, respectively, compared to the scenario that considers the management of all patients with SOC. Results are reported graphically in Fig. 2.

In case the analysis considers a control visit every three months for both SOC and FE_NO_ strategies, the savings would be 11,396,609€ and 22,793,218€ for the scenarios considering 50% FE_NO_ vs. 100% SOC and 100% FE_NO_ vs. 100% SOC, respectively.

**Table 2:** Costs detail for the different categories

Cost Category	Strategy SOC	Strategy FE_NO_	Difference %
*Exacerbations*			
Hospitalizations for serious exacerbations	31,648,508 €	5,380,246 €	-83%
Management of non-serious exacerbations	168,976,433 €	119,973,268 €	-29%
**Total exacerbations**	**200,624,942 €**	**125,353,514 €**	-38%
*Treatments*			
Corticosteroids for inhalation and combinations	663,200,000 €	384,656,000 €	-42%
Other drugs	317,500,000 €	317,500,000 €	0%
**Total treatments**	**980,700,000 €**	**702,156,000 €**	-28%
*Management of adverse events*			
Sepsis	28,485,156 €	19,748,806 €	-31%
Venous thromboembolism	3,812,104 €	2,642,938 €	-31%
Fractures	71,282,080 €	49,419,985 €	-31%
**Total management of adverse events**	**103,579,340 €**	**71,811,729 €**	-31%
*Visits*			
Total cost for visits	126,663,687 €	126,663,687 €	0%
Total cost for spirometries	187,649,907 €	187,649,907 €	0%
FE_NO_ test	0 €	181,394,910 €	100%
**Total visits**	**314,313,594 €**	**495,708,504 €**	58%
**OVERALL TOTAL**	**1,599,217,876 €**	**1,395,029,747 €**	**-13%**

**Table 3:** Total yearly costs for the management of asthma patients in Italy according to different scenarios considered

Scenarios	SOC	FE_NO_	Cost SOC	Cost FE_NO_	TOTAL IMPACT ON THE NHS	Difference compared to scenario 1 (savings)
1	100%	0%	1,599,217,876 €	0 €	1,599,217,876 €	-
2	50%	50%	799,608,938 €	697,514,874 €	1,497,123,811 €	-102,094,064 €
3	0%	100%	0 €	1,395,029,747 €	1,395,029,747 €	-204,188,128 €

## Discussion

Asthma is a major noncommunicable disease affecting both children and adults, with a high impact on their families and the society as a whole. Suboptimal adherence to treatments remains a significant barrier to asthma control contributing to an increased risk of exacerbations. The identification of unsatisfactory compliance is often difficult due to reluctances in patients self-reporting and estimates on prescriptions that may not represent the real use. Non-adherence to inhaled corticosteroid use is a major challenge to successful asthma management because it can lead to inappropriate treatment escalation, particularly in severe disease. In patients with Type-2 inflammation, FeNO showed a role in the assessment and monitoring of adherence to inhaled corticosteroids. In particular, an elevated FeNO may be a useful instrument to predict the likelihood of response to inhaled corticosteroids and risk of future exacerbations [31, 32] therefore the management of patients based on FeNO could optimize treatment prescriptions allowing the most adequate management of patients with asthma [33].

The present study estimated the overall economic impact of an extensive use of FeNO testing in the Italian population, and compared it to the management of patients according to SOC. In the baseline analysis, an increased utilization rate of FeNO testing from 50 to 100% of patients may lead to savings for the NHS from about 102 to 204 million € compared to the management of patients with SOC. Considering an increased frequency of visits (every 3 months instead of 6 months) the savings would become about 11 and 23 million € in the two considered scenarios, respectively.

The study has some limitations that need to be disclosed. First, according to the clinical practice reported by KOLs, we considered in the model a frequency of one specialist visit every 6 months for both SOC and FeNO testing strategies. The considered RCTs on FeNO benefits report shorter schedules, in particular, Smith and colleagues [21] set the visit frequency at 3 months for the evaluation of ICS dose reduction, Green et al. [19] when reported the control on serious exacerbations used a frequency of 9 visits per year (approximately every 1.3 months) while Jayaram and colleagues [20] who assessed the reduction of exacerbations used a frequency of one visit every 3 months. All the KOLs involved in the study agreed about the non-linear relationship between the number of FeNO measurements and the effectiveness of the test in optimizing the treatment, therefore, the 6-month timing considered in the model may be anyway considered representative of the clinical practice and not invalidating the FeNO test benefits.

A second limitation relates to the management of severe asthma patients who in about 70% of cases present comorbidities [34]. The analysis excluded the cost component for the management of concomitant diseases, anyway the latter should have an impact only on the frequency of hospital accesses, for example more admissions for patients with more comorbidities, and not on the economic value of the hospitalization. In fact, a comorbid asthmatic hospitalized patient is not treated differently from an asthmatic patient without comorbidity and the reimbursement applied to the hospital admission is the one already considered in the analysis. This aspect, has been totally captured by the population we considered in the analysis, which derives from estimates on administrative databases.

One of the advantages of FeNO testing is to discriminate refractory Type- 2 high patients that are likely to require personalized therapies like biologics [35]. In Italy there are published data on the consumption and expense of biologics but these figures are not disaggregated for the different specific indications (for example data on dupilumab are referred to severe atopic dermatitis, severe and refractory asthma and chronic rhinosinusitis with nasal polyps), therefore an analysis on the number of patients treated with biologics for asthma according to a FeNO test was not feasible. Moreover, the investigation is complicated by a not homogeneous diffusion of the test across the different Italian Regions.

In the analysis we gave an overview of the costs for the management of patients with asthma for both SOC and FeNO testing scenarios in Italy by combining different sources of data. The number of serious exacerbations per year requiring hospitalization was derived from two different sources reporting data at national level for patients older than 15 years and younger than 14 years, therefore there was a lack of information for patients aged 14–15 years. As the number of non-serious exacerbations was estimated by the difference between the total estimated exacerbations and the severe ones, the exacerbations for patients aged 14–15 years were classified as non-severe, thus underestimating the real management cost. Moreover, the analysis considered only specialist visits performed by the patients leading to another possible cause of underestimation of costs.

Another point relates to the effects of the determination of FeNO on the treatment optimization leading to a decrease in the frequency of exacerbations, consumption of oral corticosteroids and, therefore, the appearance of complications resulting from the use of these drugs. In this context a therapeutic adherence is assumed, anyway patients’ compliance is a complex phenomenon that is difficult to measure and might only partially depend on the use of FeNo test for asthma control [36–38]. A limited therapeutic adherence may decrease the advantages of FeNO testing.

Again, the model does not stratify patients according to confounding factors that may affect FeNO levels. Studies have shown that FeNO increases with age in children [39] and with persistent and/or high allergen exposure [40]. Olin and colleagues noted that FeNO was positively correlated with height in both males and female adults [41]. Moreover, smoke may modify airway inflammation and reduce FeNO levels, thus possibly compromising the diagnostic value of FeNO itself [42, 43].

Only few studies evaluated the cost of asthma in Italy. Dal Negro and colleagues [44] investigated the clinical data and healthcare use of 817 asthma patients of different severity through a prospective study reporting a mean cost for the NHS of 1,055€ per patient per year, including the management of comorbidities. At European level the NHS cost for the management of an asthmatic patient was estimated in 594€ per year according to an analysis of 462 patients with persistent asthma (including Italian subjects) [45]. The mean cost per patient per year for SOC of about 409€ from our study may be considered a coherent estimate taking into account the limitations of the cost assessment described above.

Another issue is related to the population considered in the studies assessing the efficacy of FeNO test; the trials evaluating the ICS dose reduction [21] and the risk reduction of exacerbations [20] considered individuals with chronic asthma in general, so an analysis evaluating the benefits of FeNO testing on a more severe population of patients with asthma was not practicable.

Despite these limitations, the present study provided a detailed analysis of the different categories to assess the cost for the management of Italian patients with asthma with SOC or with an increased utilization of FeNO testing. The study showed the advantages of FeNO testing for the optimization of the treatment for patients but also highlighted the lack of detailed data for few cost items (e.g., consumption of biologics for asthma) to perform more specific analyses. The information provided may be useful to improve the management of asthmatic patients at national level and suggest the implementation of registries for the prospective collection of clinical outcomes and healthcare resource consumption on a large scale to allow more precise analyses in the future. Moreover, the integration between hospitals and healthcare services provided at a local level is certainly essential in order to ensure continuity of care and the optimization of the services offered to patients.

## Data Availability

All data generated or analyzed during this study are included in this published article.
